# Benzo(a)pyrene and cardiovascular diseases: An overview of pre-clinical studies focused on the underlying molecular mechanism

**DOI:** 10.3389/fnut.2022.978475

**Published:** 2022-08-04

**Authors:** Chenghao Fu, Yuemin Li, Hao Xi, Zemiao Niu, Ning Chen, Rong Wang, Yonghuan Yan, Xiaoruo Gan, Mengtian Wang, Wei Zhang, Yan Zhang, Pin Lv

**Affiliations:** ^1^Department of Cell Biology, Cardiovascular Medical Science Center, Key Laboratory of Neural and Vascular Biology of Ministry of Education, Hebei Medical University, Shijiazhuang, China; ^2^Hebei Key Laboratory of Forensic Medicine, College of Forensic Medicine, Hebei Medical University, Shijiazhuang, China; ^3^Eco-Environmental Monitoring Center of Hebei Province, Shijiazhuang, China; ^4^Hebei Food Safety Key Laboratory, Hebei Food Inspection and Research Institute, Shijiazhuang, China

**Keywords:** Benzo(a)pyrene, cardiovascular diseases, AhR, oxidative stress, inflammation, genotoxicity

## Abstract

Benzo(a)pyrene (BaP) is a highly toxic and carcinogenic polycyclic aromatic hydrocarbon (PAH) whose toxicological effects in the vessel-wall cells have been recognized. Many lines of evidence suggest that tobacco smoking and foodborne BaP exposure play a pivotal role in the dysfunctions of vessel-wall cells, such as vascular endothelial cell and vascular smooth muscle cells, which contribute to the formation and worsening of cardiovascular diseases (CVDs). To clarify the underlying molecular mechanism of BaP-evoked CVDs, the present study mainly focused on both cellular and animal reports whose keywords include BaP and atherosclerosis, abdominal aortic aneurysm, hypertension, or myocardial injury. This review demonstrated the aryl hydrocarbon receptor (AhR) and its relative signal transduction pathway exert a dominant role in the oxidative stress, inflammation response, and genetic toxicity of vessel-wall cells. Furthermore, antagonists and synergists of BaP are also discussed to better understand its mechanism of action on toxic pathways.

## Introduction

BaP is a crystalline, aromatic hydrocarbon consisting of five fused benzene rings found in coal tar with the formula C_20_H_12_ in the nineteenth century (1). The main sources of BaP in food are from pollution materials in the environment or produced by the pyrolysis of amino acids, fatty acids, and carbohydrates (2).The BaP is formed during the incomplete combustion of organic matter at temperatures between 300 and 600°C, and is primarily found in automobile emissions, cigarette smoke, coal tar and charcoal-broiled foods (3, 4). Due to their lipophilic and hydrophobic characteristics, polycyclic aromatic hydrocarbon (PAH) finally accumulates in the food chain. Dietary exposure accounts for more than 90% of the total exposure to PAHs in the general population in various countries (5). Nowadays, BaP is the first pollution indicator of PAHs in food designated by the scientific community (6).

Cardiovascular diseases (CVDs) are associated with DNA damage, including DNA adduct and oxidative DNA damage, in both circulating and vessel-wall cells. And environmental chemical carcinogens have been identified to be as a risk factor for CVDs (7–10). In addition, collective evidence from these studies revealed that the cardiovascular toxicity of foodborne contaminants is mainly attributed to PAHs, especially BaP. However, no literature review focuses on the association between BaP and CVDs. In this review, we aimed to provide a comprehensive understanding of the mechanism of cell toxicity effects of BaP in CVDs, mainly focusing on atherosclerosis (AS), hypertension (HTN), and abdominal aortic aneurysm (AAA).

## Sources, metabolism and tissue toxicity of BaP

The primary sources of PAH contamination can be divided into two groups: anthropogenic pollution and natural pollution. Natural sources of PAHs are negligible or less important. The significant determinants of PAH pollution are anthropogenic pollution sources, classified into four groups, i.e., industrial, mobile, domestic, and agricultural pollution sources (11). In fact, the main source of exposure to PAHs for non-smokers and non-occupationally exposed adults are food. Diet contributes to more than 90% of total PAHs exposures in the general population in various countries (2, 5). PAHs can generate during the food preparation procession (contaminated by environmental PAH that are present in air, soil, or water by deposition and transfer), or during processing and cooking (12). BaP is the first pollution indicator of PAHs in food. Therefore, foodborne BaP contaminants are a primary source of BaP uptake by humans.

After entering the body, except for a small part of BaP excreted in the feces in its original form, most of the BaP accumulated in the gastrointestinal tract, epididymal fat, lung, liver, brain, and kidney (13). BaP is highly lipophilic and can be easily absorbed into cells through the plasma membrane. BaP can be metabolized into dozens of metabolites through AhR and aromatic hydrocarbon metabolizing enzymes (14–16), such as 1, 2-dihydroxy-1, 2-dihydrobenzopyrene, benzopyrene diketone, and BPDE. The conversion to hydroxyl compounds or ketones is a detoxification reaction, while the conversion to epoxide is an activation reaction. About 10% of BaP is converted to BPDE in the organism (17). BPDE has a strong oxidizing capacity, which can cause oxidative damage to DNA, affect DNA replication, and induce apoptosis and gene mutation.

BaP is well known for its carcinogenic activity early in 1930, and numerous studies since the 1970s have documented links between BaP intake and cancers (1, 18, 19). It has been classified as class I carcinogen by the International Agency for Research on Cancer (www.iarc.who.int). The exposure of BaP results in extensive and selective formation of BPDE, which has high interaction activity with DNA and forms a DNA adduct, the major mutagen in tobacco smoke (7, 20). Properly speaking, BaP is a procarcinogen. Its carcinogenic effects depend on the activity of the detoxification enzymes cytochrome P450 1A1 (CYP1A1) and CYP1B1, which enzymatic metabolism BaP to BPDE (21, 22). Furthermore, BaP induces the CYP1A1 gene expression by activating the AhR nuclear translocation signal pathway (23, 24). Furthermore, most of the BPDE-DNA adducts can be removed from DNA by nucleotide excision repair mechanism within the cell. Therefore, continuous or high doses of BaP intake will inevitably cause errors during DNA replication leading to carcinogenic mutations (23–25). Besides, there is growing evidence that BaP has strong toxic effects on the nervous system (26), immune system (27, 28), and reproductive system (29).

AhR is a ligand-activated transcription factor best known for mediating carcinogen toxicity and tumor-promoting properties, including dioxin and BaP (30). AhR belongs to the basic helix-loop-helix transcription factors family. And numerous studies have revealed that the toxicity of BaP has been linked to activation of the AhR (31–33). In the absence of ligands, AhR exists predominantly in the cytosolic compartment in association with a chaperone complex (Hsp90/XAP/p23) (30). Upon BaP binding to AhR, dimerization of AhR and the aryl hydrocarbon receptor nuclear translocator (ARNT) occurs. The AhR/ARNT heterodimer then binds to xenobiotic responsive elements (XREs) (core sequence: GCGTG) in the promoters of BaP-regulated genes, such as cytochrome P450s (CYPs), intercellular cell adhesion molecule 1 (ICAM1), vascular cell adhesion molecule 1 (VCAM1), and prostaglandin endoperoxide synthase 2 (PTGS2) (33–35).

CYPs are membrane-associated proteins that use molecular oxygen and reduce the equivalents of NADPH to catalyze oxidative, peroxidative, and reductive metabolism of endogenous and exogenous substrates (36). More than 400 genes encoding CYP-associated activities have been cloned, but their relative expression exhibits remarkable tissue, gender, and developmental specificity (37). Kerzee and Ramos investigated constitutive and inducible expression of these two CYPs from AhR knockout mice. Their results show that the expression of CYP1A1 was inducible in BaP-treated AhR^+/+^ mice, and CYP1B1 was expressed under constitutive and inducible conditions irrespective of AhR phenotype or growth status (36). In mouse aortic smooth muscle cells, BaP increased the aryl hydrocarbon hydroxylase activity. The specific inhibitor of CYP1B1, but not CYP1A1, could reverse the BaP-induced DNA adducts formation, which may contribute to atherogenesis by PAHs (38). Furthermore, CYP1A1^−/−^ and AhR^−/−^ mice formed smaller atherosclerotic lesions size and oxidative stress when suffering over 10 weeks of 10 mg/kg/body weight(bw) BaP treatment (39, 40).

## Vascular toxicity effects and underlying mechanism of BaP

As early as 1977, it was reported that the aorta was the target organ of BaP (41). However, it is only in recent years, the cardiovascular toxicity of BaP attracted considerable attention. Currently, as an independent risk factor for CVDs, BaP has been found to be closely related to CVDs, including AS, HTN and AAA, and shows multiple kinds of vascular toxicity (10, 11, 13, 42). Preclinical studies have found that BaP exposure is correlated with oxidative stress and vascular toxicity. In addition, investigations have discovered a relationship between BaP exposure and the occurrence and development of CVDs (11).

### Atherogenic effect of BaP

Atherosclerosis (AS) is a chronic pathological process in the large artery wall and is characterized by the accumulation of oxidized lipid, fibrous elements, and calcification. This process is initiated by endothelium injury, followed by a cascade of events, which causes the vessel narrowing and activation of inflammatory responses leading to atheroma plaque formation (43–45). As a result, these processes result in multiple cardiovascular complications, including myocardial infarction, heart failure, stroke, and claudication, which remain the leading cause of death globally (46). Moreover, this complex disease is caused by the interaction of multiple genetic and environmental risk factors, which include western pattern diet, tobacco smoking, and air pollution (45).

In ApoE^−/−^ background atherosclerotic animal models, 4 days of 5–8 mg/kg/bw BaP grave treatment can increase the serum epsilon A and high-density lipoprotein (HDL) level (47). Moreover, the BPDE-DNA adduct could also be observed in the aorta tissues (47), while compared to the control groups, 12–24 weeks of treatment increased the expression of monocyte chemoattractant protein 1 (MCP1), and promoted the release of transforming growth factor beta (TGFβ) and tumor necrosis factor α (TNFα) in vascular wall cells (48–50). In response to the inflammatory mediator, the atherosclerosis lesion may be heavily infiltrated with pro-inflammation cells, including macrophages, T cells and neutrophils (49), and the lesion size began to enlarge (13, 50).

Exposure to BaP plays a vital role in the etiology of atherosclerosis (Table 1). The endothelium represents the inner cell layer of blood vessels and is supported by smooth muscle cells and pericytes, which form the vessel structure (51). Due to direct blood contact, the blood vessel endothelium is inevitably exposed to genotoxic substances that are systemically taken up by the body, including BaP (17). One key step in the development of atherosclerosis is vascular endothelial dysfunction, followed by cell death and a local inflammatory response (48, 53, 67). Besides, there is strong evidence to suggest oxidative stress is one of the most potent inductors of vascular inflammation in atherogenesis. nuclear factor-κB (NF-κB) may respond directly to BaP-induced oxidative stress, and the activation of NF-κB is a key redox-sensitive event associated with vascular dysfunction (54).

**Table 1:** Effects of BaP on AS.

	**Models**	**Treatment**	**Effects**	**Reference**
Cell model	HUVECs	1 μmol/L BaP for 2 h	pro-inflammation and enhance COX2, CYP1A1 and cPLA2 activity; ↑ CYP1A1, ICAM1, VCAM1, ↓PTGS2, PLA2G4A, NOS3 gene expression	(51)
		10–25 μmol/L for 24 h	↑ monocyte adhesion and ICAM-1 depend on AhR activation, ↑ MEK, p38-MAPK, c-Jun phosphorylation; ↑ AP-1 DNA binding	(52)
		0.5–1.5 μmol/L BPDE for 96 h	↑ apoptosis, necrosis, ↓ ERCC1, ERCC4 and ligase I, ↑ BPDE-DNA adducts	(17)
		0–10 μmol/L for 4 or 24 h	↑ MCP1, CYPIA1, ↓ cell viability	(48)
		10 μmol/L for 1–5 d	↑ VEGF, and can be reversed by ERK inhibitor	(53)
		10 μM for 24h	↑ CCL1, CYP1A1 in an AhR- and calcium-dependent manner	(32)
	Human endothelial progenitor cells	10-50 μmol/L for 24 h	↓ proliferation, migration, adhesion, and angiogenesis, ↑ IL1β, TNFα, ROS, ↑ NF-κB activation	(54)
		0.1–10 μmol/L for 5–7 d	↓ adherent and proliferation, ↑ CYP1A1, and reversed by AhR antagonist, ↑ PAH-related adducts	(55)
	Human fetoplacental ECs	0.01–1 μmol/L for 6–24 h	↓ angiogenesis, ↑ COX2, PTGS2 mediated by AhR activation	(33)
	Human coronary artery ECs	30 μmol/L for 0–140 min	↑ 3H-arachidonate release and apoptosis, ↑ phospholipase A2 activation	(56)
	Mouse aortic endothelial cells	1 μmol/L	↑Cu/Zn- SOD and catalase, ↑AhR, CYP1A1/1B1 protein level; ↑ GST activity and BaP detoxification;	(31)
	Rat VSMCs	10 μmol/L for 24 h	↓ NO-induced apoptosis, ↑ NF-κB and MAPK, ↑ IL6 production	(57)
		0.1–2 μmol/L for 24 h	↑ cell migration and invasion, ↑ MMPs, and inhibited by MMPs inhibitor or AhR antagonist	(58)
		0–10 μmol/L for 0–30 h	↓ T-cadherin, and reversed by AhR antagonist a-naphthoflavone	(59)
		0.1–5μmol/L for 24 h	↑ COX2, prostaglandin, ERK phosphorylation, and NF-κB activation; reversed by MAPK or NF-κB inhibitor	(60)
		3 μmol/L for 24 h	↑ C/EBP-α/β, ARE/EpRE repressed, whereas AhR enhanced, GST-Ya gene expression	(61)
	Mouse VSMCs	3 μmol/L for 24 h	↑ DNA adducts, ↑ aryl hydrocarbon hydroxylase and CYP1B1 activity	(38)
		3 μmol/L for 1–5 h	↑ CYP1A1, CYP1B1 and reversed by AhR knockout	(36)
		0.03–3 μmol/L for 24 h	↑ ROS, ARE/EpRE, ↓ c-Ha-ras transcription	(62)
		0.3–2μmol/L for 1–5 h	↑ c-Ha-ras and oxidative stress; inhibited by P450 or AhR inhibitor ellipticine	(63)
		10 μM for 24 h	TGFβ2 and IGF1 are potential candidates signaling pathways of AhR	(64)
	HAECs, HCSMCs	3μmol/L for 24 h	↓ prolyl-4-hydroxylase, ↓ cellular collagen levels, atherosclerotic cap thickness	(65)
Animal models	ApoE^−/−^ mice	5 mg/kg/bw daily for 4 d	↑ aorta BPDE-DNA adduct, epsilon A, and HDL level	(47)
		5 mg/kg/bw, weekly for 2 w	↑ aortic tissue MCP1 gene expression	(48)
		5 mg/kg/bw, weekly for 12–24 w	↑ plaques and lipid core size; ↑ T cells and macrophages infiltration;	(49)
		5 mg/kg/bw, weekly for 24 w	↑ PAH-DNA adducts in lung, ↑ TGFβ and TNFα release, ↑ atherosclerotic plaque size	(50)
		8.5 mg/kg/bw daily for 24 w	↑ inflammatory response, ↑ atherosclerosis lesion size	(13)
	ApoE^−/−^ mice; CYP1A1^−/−^ mice	12.5 mg/kg/day	↑ atherosclerotic lesions, ↑ ROS level, ↑ inflammatory markers; ↑ VEGF gene expression, ↑ DNA adduct formation	(39)
	ApoE^−/−^;AhR^b1/b1^ and ApoE^−/−^;AhR^d/d^	10 mg/kg/bw, 5 days/week for 10–23 w	↑(↓) plaque size and initial time, ↑(↓) AhR affinity, ↑(↓) immune response genes	(40)
	ApoE^−/−^;hSod1^−/−^ mice	2.5 mg/kg/bw weekly for 24 w	↑ oxidized lipids, ↑ atherosclerotic lesions; and ↓ cell adhesion molecules, monocyte adhesion, ↓ oxidized lipids, ↓atherosclerotic lesions	(66)

“↑” means up-regulation and “↓” means down-regulation; human aortic endothelial cells (HAECs); human coronary artery smooth muscle cells (HCSMCs); unless noted the treatment agent is BaP in all Tables.

Caveolae are non-clathrin-coated plasma membrane microdomains enriched in cholesterol and glycosphingolipids. They are particularly abundant in endothelial cells and play an important role in membrane traffic and cellular signal transduction (52, 68). Oesterling and his collage observed that caveolin-1 mediated the BaP-induced ICAM1 expression in primary human endothelial cells. They also illustrated that β-naphthoflavone/BaP induced ICAM1 expression by signaling through MEK, MAPK, and AP-1 leading to increased adhesion of monocytes to the activated endothelium (52, 68).

Besides, BaP-induced bulky DNA adducts and the consequent DNA mutations in vascular cells are considered to be involved in AS (31). BaP forms BPDE through a three-step activation process catalyzed by human cytochrome P450 enzymes, notably CYP1A1 and CYP1B1, and by epoxide hydrolase (17). A screening for DNA repair factors in BPDE treated human umbilical vein endothelial cells (HUVECs) revealed that the nucleotide excision repair (NER) proteins excision repair cross-complementation (ERCC) 1, ERCC 4 and ligase I were expressed at lower levels in HUVECs compared with human umbilical artery smooth muscle cells (HUASMCs) and haemopoietic progenitor cell (HPCs), which corresponds with the impaired NER-mediated removal of BPDE adducts from DNA (17). These data revealed that HUVECs is more sensitive to BPDE than HPCs and HUASMCs and displays an unexpected DNA repair-impaired phenotype.

Vascular smooth muscle cells (VSMCs) are located in the mid-membrane of the vascular wall and are responsible for the structural characteristics of the vessel wall. Abnormal proliferation, migration, and invasion of VSMCs have been suggested to be the major contributor to the development of atherosclerotic lesions (57, 58, 69). Evidence shows that BaP could activate interleukin 6 (IL6) production and suppress nitric oxide-induced apoptosis in VSMCs. A significant role of IL6 in the pathophysiology of atherosclerosis has also been suggested, and atherosclerosis even has been suggested to be an inflammatory disease (69, 70). Furthermore, BaP was capable of inducing the activation of NF-κB and MAPK in VSMCs. Both NF-κB inhibitor and MAPK inhibitor significantly reversed the anti-apoptotic effect of BaP on NO-induced VSMCs apoptosis (57).

Matrix metalloproteinases (MMPs) are a family of over 20 different endopeptidases that each degrades several extracellular matrix proteins and non-matrix substrates. MMPs play a major role in cell migration, differentiation, angiogenesis, and host defense (71). Expression of various MMPs was found to increase in BaP-induced transcriptional activation of MMPs, especially MMP3, is not through activator protein 1 (AP-1) or NF-κB, and the expression of MMPs increased the migration and invasion ability of VSMCs in rats (58). Besides, when treated with BaP, the expression level of T-cadherin, an atypical glycosylphosphatidylinositol-anchored member of the cadherin superfamily of adhesion molecules, is significantly repressed (59). However, further investigation of the relationship between the upregulation of MMPs and T-cadherin degradation still needs further exploration.

Cyclooxygenase (COX2), also known as prostaglandin-endoperoxide synthase (PTGS2), is a rate-limiting enzyme responsible for prostaglandins forming and plays both physiologic and pathologic roles in vascular function. In BaP-treated VSMCs, ERK and NF-κB signal pathways are involved in the expression of COX2, which may participate in the genesis of AS (60).

Miller and his colleagues observed that treatment of VSMC with BaP induced reactive oxygen species (ROS) accumulation which leads to a variety of different outcomes, including activation of nuclear proteins to bind antioxidant response element/electrophilic response element (ARE/EpRE), activation of cytosolic proteins that translocate and bind ARE/EpRE, or redox sensor that interacts with cellular proteins to activate binding to ARE/EpRE. As a result, oxidative intermediates of BaP mediate activation of nuclear protein binding to ARE/EpRE and contribute to transcriptional de-regulation of c-Ha-ras (62). Kerzee et al. (63) found the upregulation of oxidative stress and c-Ha-ras in VSMCs could be reserved by CYPs and AhR inhibitor ellipticine. Chen et al. (61) observed another nuclear protein CCAAT/enhancer-binding protein alpha and beta (C/EBP-α, β) which was activated by AhR signal pathway and lead to the inhibition of glutathione S-transferase (GST)-Ya subunit gene expression. In addition, overexpression of antioxidant enzymes suppressed BaP-accelerated atherosclerosis in ApoE-deficient mice (66, 72).

### BaP and hypertension

Hypertension (HTN), also known as high blood pressure, is a long-term medical condition in which the blood pressure in the arteries is persistently elevated (7). HTN usually does not cause noticeable symptoms. However, long-term untreated HTN is a major risk factor for heart attacks, stroke, atrial fibrillation, heart failure, and peripheral arterial disease. HTN is a major cause of premature death worldwide (73, 74). Over 90% of HTN is classified as a primary type and is usually caused by unhealthy lifestyles such as a high salt diet, overweight, alcohol drinking, and smoking. The remaining cases are categorized as secondary types due to identifiable causes, including chronic kidney disease, narrowing of the kidney arteries, and an endocrine disorder (73).

Studies have shown that systolic blood pressure is significantly increased, and aortic responsiveness to phenylephrine is enhanced in rats exposed to BaP (Table 2). Inhibitors of protein kinase C (PKC), MAPK, myosin light-chain kinase (MLCK) and Rho kinases significantly inhibit BaP-enhanced vasoconstriction (Table 2). BaP induces ROS production in vascular smooth muscle cells in a time-dependent manner (7). Heart rate was not affected in BaP-treated sprague-dawley rats, however, weight loss, markedly elevated blood pressure (14). BaP exposure may affecte cardiovascular development and increased systolic blood pressure. Jules et al. (76) show that exposure to BaP results in functional deficits in offspring during cardiovascular development, which may lead to cardiovascular dysfunction later in life. BaP exposure altered the circadian pattern of blood pressure, with a reduction in the normal dipping pattern during sleep. This was associated with increased neutrophil recruitment in the lungs of BaP-exposed rats (75). Intraperitoneal injection of BaP up regulated the expression of CYP1A, CYP1B1, CYP1C1, CYP1C2, and COX1 in zebrafish mesenteric arteries suggesting that BaP is associated with changes in cardiovascular function (77).

**Table 2:** Effects of BaP on HTN and AAA.

	**Models**	**Treatment**	**Effects**	**Reference**
Hypertension	Rat aortas and VSMCs	1–10 μmol/L BaP	↑ vasoconstriction and reversed by AhR, PKC, MAPK, MLCK, and Rho-kinase inhibitor; ↑ ROS	(7)
	Sprague-Dawley rats	20 mg/kg/bw for 8 w	↑ systolic blood pressure, ↑ aortic hyperreactivity to phenylephrine	(7)
		0.01 mg/kg, Intranasal	altered rhythm of blood pressure, ↑ lung neutrophil recruitment	(75)
		0.15–1.2 mg/kg/ bw at E14-17	↑ blood pressure relative genes NOS, eNOS, NADP oxidoreductase (BH4/BH2) and AngII	(76)
		10 mg/kg/bw, weekly for 4 w	↑ blood pressure	(14)
	Zebrafish	1 mg/kg/bw for 24 h	↑ CYP1A, CYP1B1, CYP1C1, CYP1C2, and COX-1 in mesenteric artery	(77)
Abdominal Aortic Aneurysm	WT mouse +Ang II	10 mg/kg/bw, weekly for 6 w	↑ AAA pathogenesis, ↑ VSMC apoptosis	(78)
		10 mg/kg/bw, weekly for 5 w	↑ AAA incidence, ↑ macrophage infiltration, elastic lamella degeneration	(79)
		10 mg/kg/bw, weekly for 5 w	↑ AAA pathogenesis, ↑ macrophage infiltration, ↑ MMP2, MMP9, MMP12, NF-kB expression	(80)
	ApoE-/- mouse+Ang II	5 mg/kg/bw, weekly for 7 w	↑ AAA pathogenesis, ↑ TNFα, Cyp1 A, MMP9	(42)
Myocardial injury	Sprague-Dawley rats	0–10 μmol/L BaP for 0–48 h	↑ ROS, ↑ NCF1/p47(phox) in macrophages, and reversed by AhR knock down	(81)
		20 mg/kg/bw for 7 d	↑ cardiac hypertrophy, ↑ CYP1A1, CYP1B1, CYP2E1, CYP4F4, CYP4F5 and soluble epoxide hydrolase	(82)
	Zebrafish embryos	5 μmol/L	↑ cardiac abnormalities, ↑ CYP1A1,	(83)
		100 μg/L for 2–18 h	↑cardiac deformities, Ca2+-cycling gene alteration	(84)
		0.02–2 μmol /L for 72 h	↑cardiotoxicity, ↑ AhR1B, CYP1C1, CYP1A1, MMP9, ↓ prox1, tbx5, pak2a	(85)
		5,000 ng/L for 5 d	↑ cardiac deformities, ↑ CYP1A, ↓ COX2b	(86)
Angiogenesis	HUVECs	0.5 μmol/L (BPDE)	↓ angiogenesis, ↓ Notch1, ↑ Dll4, Jag1, and ↓ Hey2	(10)
		0–10 μmol/L for 24 h	↓ angiogenesis, ↓ integrin αv/β3, AhR, MAPK phosphorylation, ↑ CYP1A1	(87)
	Zebrafish embryos	1 μmol/L for 24–96 h	↑ cardiovascular toxicity, ↓ AhR2, myh6, ↑ CYP1A, atp2a2	(88)
	Japanese medaka	0.1–1 μg/L for 6 d	↑ heart hypertrophy, ↑ CYP2J23, Coro2A	(89)
	WT and AhR-null mice	125 mg/kg/bw weekly for 4 w	↑ ischemia-induced angiogenesis, ↑ IL6, VEGF in AhR-null mice	(90)
	Kunming mice	0.2–20 mg/kg/ bw for 1–8 d	↓ decidual angiogenesis, ↓ CD34, ER, FOXO1, HoxA10, and BMP2	(91)
	Sprague-Dawley rats	0.2 mg/kg/bw for 9 d	↓ luteal angiogenesis and vascular maturation, ↓ VEGFR, Ang-1 and Tie2, ↑ THBS1	(10)

“↑” means up-regulation and “↓” means down-regulation.

### BaP and abdominal aortic aneurysm

Abdominal aortic aneurysm (AAA) is defined as a localized enlargement of the abdominal aorta more than 50% of its diameter. The aortic wall continues to weaken and becomes unable to hold the forces of the luminal blood pressure, resulting in progressive dilatation and rupture. They usually cause no symptoms, except during rupture. Smoking and advanced age are the primary risk factors for AAA; if ruptured, the mortality is 85−90% (42, 92).

Co-stimulation of male C57BL/6J mice with angiotensin (Ang) II and BaP induced AAA significantly increased rates of formation and mean aortic diameter. The samples were subjected to circRNA expression analysis, and a circRNA-miRNA co-expression network was established based on six apoptosis-related circRNAs. Genes regulated by this network map to multiple pathways, including apoptosis, IL-17 signaling, and vascular endothelial growth factor signaling, all of which are involved in AAA formation (78). BaP increases macrophage infiltration, activates NF-κB, upregulates MMP2, MMP9, and MMP12 expression, elastic lamina disorder, and VSMCs loss (79), which increased AAA formation and rupture in C57/B6J mice (80). Furthermore, the metabolites of BaP such as 7,8-dihydrodiol, 3,6-, and 6,12-dione metabolites are reported involvement in BaP induced abdominal aortic toxicity *via* elevating plasma ROS levels and increased protein expression of TNFα, CYP1A1, and MMP9 (42).

### BaP and myocardial injury

In BaP-treated offspring rats, microarray and quantitative real-time PCR analysis revealed that the up-regulated gene expression of AngII, angiotensinogen and, eNOS, which are associated with the dysregulate cardiovascular development (76). BaP increased heart-to-body weight ratios, as well as hypertrophy markers, atrial natriuretic peptide and brain natriuretic peptide. BaP treatment increased the gene expression of CYP1A1, CYP1B1, CYP2E1, CYP4F4, CYP4F5 and soluble epoxide hydrolase. BaP treatment increased the ratios of dihydroxyeicosatrienoic acid and 20-hydroxyeicosatetraenoic acid in total epoxyeicosatrienoic acid. Benzo(e)pyrene, an isomer of BaP and a poor ligand for AhR, did not cause cardiac hypertrophy in rats, confirming the role of AhR in the development of cardiac hypertrophy (82). Zebrafish exposed to solutions containing 5 μmol/L BaP treatment exhibit cardiovascular malformations. Microarray analysis was performed to identify heart-specific transcriptomic changes in BaP/fluoranthene (FL) during early development, with Ca^2+^ cycling and muscle contraction genes being the most differentially expressed class of transcripts (Table 2). BaP/FL may affect cellular Ca^2+^ levels, which subsequently affect myocardial function and may underlie BaP/FL cardiotoxicity (84). Exposure of zebrafish embryos to BaP for 72 h resulted in defective cardiac development in zebrafish embryos (85). BaP co-exposure with alpha-naphthoflavone or resveratrol resulted in the most dramatic changes in heart and vessel morphology, with decreased ventricular length and width, increased ventricular wall thickness, and increased vessel lumen diameter. In addition, decreased expression of COX2, which is inversely associated with cardiac malformations and vasodilation (86). Moreover, BaP-induced NCF1/p47(phox) expression enhances superoxide anion production in an AhR-dependent manner in PMA-treated human macrophages; regulation of NCF1/NADPH oxidase by such PAHs may be involved in atherosclerotic heart disease associated with vascular disease such as sclerosis (81).

### BaP and angiogenesis

BaP exposure is also associated with angiogenesis both *in vivo* and *in vitro* (Table 2). Evidence shows that BaP reduced corpus luteum number, disrupted steroid secretion, affected the corpus luteum vascular network in pregnant female mice, and significantly decreased angiogenic factors (VEGFR, Ang-1, and Tie2), and increased the anti-angiogenic factor THBS1. The BaP metabolite BPDE also interfered with the expression levels of angiogenesis-related factors, such as Notch signaling molecules in HUVECs *in vitro* (10). The expression of several decidua-related factors was altered, including FOXO1, HoxA10, and BMP2 (Table 2). BaP reduced CD34 expression, suggesting that BaP treatment inhibited decidual angiogenesis. Furthermore, BaP induced the downregulation of vascular endothelial growth factors suggesting that oral administration of BaP impairs decidualization and decidual angiogenesis (91).

After exposure to BaP, the expression of metallothionein was upregulated in the ischemic hindlimb of wild-type mice and markedly inhibited ischemia-induced angiogenesis. The mRNA amount of IL6 and VEGF was reduced in the ischemic hindlimb of wild-type mice (90). BaP-treated HUVECs reduced endothelial capillary formation, cell migration, MAPK phosphorylation, and integrin expression when stimulated by angiogenic factors. Angiogenesis was also inhibited in the chorionic villus assay (87).

Using zebrafish embryos model, 6H-benzo[cd]pyren-6-one induced developmental and cardiovascular toxicity at lower doses, including reduced heart rate and blood flow. The mixture of PAHs and oxy-PAHs may lead to increased developmental and cardiovascular toxicity of zebrafish embryos through an AhR-dependent mechanism (88). Embryonic teratogenicity and developmental toxicity of BaP in Japanese medaka, BaP was efficiently incorporated into embryos by nanosecond pulsed electric field treatment. Embryos containing BaP exhibited typical teratogenic and developmental effects, such as cardiovascular abnormalities, dysplasia, and spinal curvature (89).

### BaP and other CVDs

Two blood-testis barrier proteins, Claudin-11 and Connexin 43, were impaired by treatment with a mixture of 1 μg/L streptozotocin and 1 μg/L BaP (93). The function of blood brain barrier was assessed by measuring the transendothelial electrical resistance of mouse brain microvascular endothelial cells, and the viability of cells was altered in the presence of BaP (94). Besides, BaP elicited mouse testicular sertoli cells apoptosis and blood-testis barrier disruption, which involves mitochondrial dysfunction and oxidative stress (95). These evidence imply that BaP can damage vascular barrier which in turn cause other injury includes neurotoxicity and reproductive toxicity.

Chronic BaP exposure did not alter hepatocellular carcinoma cell (HCC) growth, but promoted cell migration and invasion both *in vitro* and *in vivo*. There was a negative correlation between BaP exposure and survival in tumor-bearing mice. In addition, BaP-treated HCC cells recruited vascular endothelial cells and promoted tumor angiogenesis, possibly by increasing the secretion of vascular endothelial growth factor. The NF-κB pathway may be an adverse outcome pathway related to the cumulative effect of BaP on HCC metastasis (96).

## Antagonist and synergistic agents of BaP

Numerous reports examined different types of agents in interfering BaP metabolism and molecular signing alteration, which have potential therapeutic implications in BaP induced CVDs (Table 3). Gdula-Argasińska et al. observed that resolvin D1, a product of transcellular biosynthesis with leukocytes and endothelial cells from docosa-hexaenoic acid, reversed the overexpression of COX2, cPGES and repression of GSTM1 protein after co-cultured with BaP incubated HUVEC. Besides, an increase of cPLA2 and a decrease of CYP1A1 activity were also noted in RvD1 and BaP co-treatment (97). These data suggested that resolvin D1 could contribute to vascular function and alleviate the harmful effects caused by BaP, which might potentially aid in repairing the injured endothelium. In addition, flavonoids that contain a 40 B-ring hydroxyl substitution and a 2-3 C-ring double bond can decrease proinflammatory molecular ICAM1 expression in endothelial cells (98). Two latest reports show that bioactive compounds, hesperidin, and phaeodactylum tricornutum extracts can also inhibit the pro-inflammation mediators, including IL1β, TNFα, and IL8 (99, 100). Moreover, Budesonide-poly(lactide-co-glycolide), Soluble epoxide hydrolase inhibitor, and Ginkgo biloba extract have been proved to have anti-oxidative effects. In BaP-treated animals, the repression of ROS, NO, and CYP1A1 level and multiple cell protection effects were observed (100, 102, 103). Collectively, the above observations indicate that the anti-oxidative and anti-inflammation natural extract or inhibitors have potential protective effects on BaP-induced damage on vascular wall cells.

**Table 3:** Antagonist and synergistic agents of BaP.

	**Models**	**Treatment**	**Effects**	**Reference**
Antagonist agent	Resolvin D1	HUVECs	↓ BaP-induced CYP1A1, PTGS2, COX2, cPGES, ↑ GSTM1 level; and ↑ cPLA2, ↓ CYP1A1 activity	(97)
	Flavonoids	HUVECs	↓ BaP-induced ICAM1 expression in HUVEC	(98)
	Hesperidin	Human EAhy-926 cells	↓ BaP-induced AhR activation, ↑ ABCA1, ↓ IL-1β and TNFα, ↓ LDL accumulation	(99)
	Ostreococcus tauri and Phaeodactylum tricornutum Extracts	Human micro-vascular endothelial cell	↓ cell apoptosis and extracellular vesicles, ↓ CYP1A1, IL-8 and IL1-β	(100)
	Budesonide-poly(lactide-co-glycolide)	A/J mice	↓ BaP-induced oxidative stress, and vascular leakage, ↓ VEGF and c-myc expression	(101)
	Soluble epoxide hydrolase inhibitor	Sprague-Dawley rats	reversed the BaP-induced CYP1A1, CYP1B1, CYP4F4, and CYP4F5 ↑	(102)
	Ginkgo biloba extract	Stomach Neoplasms mice	↓ ameliorating cardiotoxic effects of doxorubicin, ↓ serum NO, ↓ liver cytosolic glutathione S-transferase, G6PDH activity	(103)
Synergistic agent	1,25(OH)2D3	THP-1 and U937 cells	↑ BaP-DNA adduct formation	(104)
	Carbon black particles	Human EAhy-926 cells	↑ cell proliferation, migration and invasion, and metabolism, ↓ PPARγ activity	(105)
	SiNPs	HUVECs	↑ ROS, DNA damage, cell cycle arrest, ↑ bax, caspase-3, and caspase-9, ↓ Cdc25C, cyclin B1, bcl-2	(106)
		Zebrafish embryos	↑ inflammation and coagulation, ↑ pAP-1/c-Jun, CD142	(107)
		Zebrafish embryos	signaling pathway alteration such as MAPK, PI3K-Akt, JAK/STAT	(108)

“↑” means up-regulation and “↓” means down-regulation.

Using the zebrafish model, Duan and Asweto et al. found that co-exposure of silica nanoparticles and BaP activate the AP-1/c-Jun and MAPK/PI3K signaling pathway, upregulate the expression of proinflammatory and procoagulant genes. As a result, silica nanoparticles help trigger the inflammation response and ROS generation, which could cause cardiac toxicity and erythrocyte aggregation (106–108). Interestingly, the protective effects of vitamin D on cardiovascular disease are principally mediated by the conversion of vitamin D to the active form, 1α,25-dihydroxy vitamin D3 [1,25(OH)2D3]. However, combined treatment with BaP and 1,25(OH)2D3 enhances BaP toxicity, including BaP-DNA adduct formation and ROS production (104).

## Conclusions

**Figure 1:**
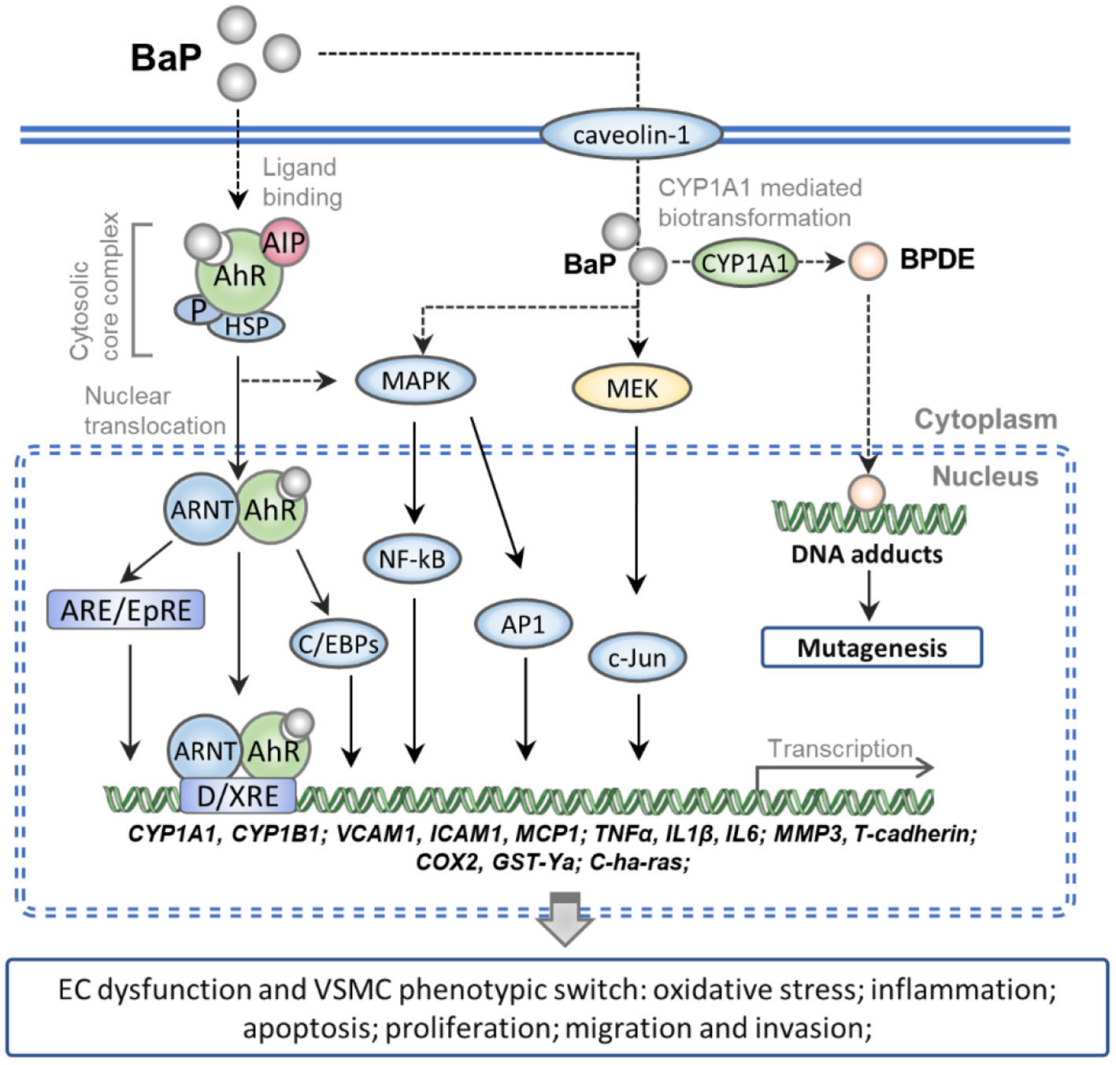
Schematic representation of the molecular mechanism of BaP exposure-induced CVDs. Numerous pieces of evidence show that the caveolin-1 can facilitate entry of the BaP into vessel-wall cells. The AhR complex is translocated into nuclear after binding with intracellular BaP. And then the BaP-AhR complex could activate the AhR/ARNT/XRE, MEK/c-Jun, and MAPK/NF-kB/AP-1 signaling pathway which can up-regulate the gene of CYPs, cell adhesion molecules, pro-inflammatory factors, and peroxidase. Besides, the activated CYP1A1 actually increased the metabolism of BaP into BPDE, which can form DNA adducts and induce mutation. As a result, BaP exposure increases the ECs dysfunction and VSMC phenotypic switch which accelerates the pathological progress of CVDs.

The present review summarizes the molecular mechanisms underlying the vascular cell toxicity effects of BaP in CVDs as follows: (1) numerous studies have demonstrated that BaP can accelerate the pathological progress of AS, and many lines of evidence show that excessive daily BaP intake is a potent risk factor which accompanied by AAA, HTN, and MI. (2) BaP binds to the ligand-activated transcription factor AhR, which evokes oxidative stress and inflammation response molecules in ECs *via* activating the AhR/ARNT/XRE, MEK/c-Jun, and MAPK/NF-kB/AP-1 signaling pathway. (3) BaP exposures increase the cell proliferation, migration, and invasion ability of VSMC by a similar signaling pathway in BaP-treated ECs. (4) The activation of CYP1A1 actually increased the genetic toxicity on vessel-wall cells through the metabolism of BaP into BPDE, which can form DNA adducts and induce mutation (Figure 1). Furthermore, compounds with AhR activation activity probability exert synergism effects on BaP vascular toxicity. In contrast, this toxicity might be alleviated by bioactive materials which process anti-oxidative and anti-inflammation action.

## Author contributions

PL and YZ: ideas, conceptualization, supervision, and funding acquisition. CF and YL: resources, literature collection, and writing-review and editing. HX, ZN, NC, RW, YY, XG, MW, and WZ: literature collection and initial draft writing. All authors contributed to the article and approved the submitted version.

## Funding

This work was supported by the National Key Research Project of China (2019YFC1606400), Major Public Welfare Projects in Henan Province (201300110200), National Key Research Project of Hebei Province (20375502D), Fund of National R&D Center for Edible Fungus Processing Technology, Henan University (20200109), Postdoctoral Research Funds of Hebei Medical University (30705010016-3759), and University Science and Technology Research Project of Hebei Province (QN2017107).

## Conflict of interest

The authors declare that the research was conducted in the absence of any commercial or financial relationships that could be construed as a potential conflict of interest.

## Publisher's note

All claims expressed in this article are solely those of the authors and do not necessarily represent those of their affiliated organizations, or those of the publisher, the editors and the reviewers. Any product that may be evaluated in this article, or claim that may be made by its manufacturer, is not guaranteed or endorsed by the publisher.
